# Measuring how healthcare organizations respond after patients experience harm: perspectives and next steps

**DOI:** 10.3389/frhs.2024.1488944

**Published:** 2025-01-21

**Authors:** Lauge Sokol-Hessner, John Adams, Carole Hemmelgarn, Beth Miller, Diane O'Connor, Melissa Parkerton, Leilani Schweitzer, J. Matthew Austin

**Affiliations:** ^1^ Division of General Internal Medicine, University of Washington, Seattle, WA, United States; ^2^ UW Medicine Collaborative for Accountability and Improvement, Seattle, WA, United States; ^3^ Independent Researcher, Philadelphia, PA, United States; ^4^ MedStar Health Institute for Quality and Safety, Columbia, MD, United States; ^5^ CommonSpirit Health, Chicago, IL, United States; ^6^ Armstrong Institute for Patient Safety and Quality, Johns Hopkins Medicine, Baltimore, MD, United States; ^7^ Risk Management Communications, Reno, NV, United States; ^8^ Department of Anesthesiology and Critical Care Medicine, Johns Hopkins University School of Medicine, Baltimore, MD, United States

**Keywords:** patient safety, quality measurement, communication, reconciliation, benchmarking

## Abstract

Patients can experience serious harm from healthcare, the impacts can be prolonged, and events may also affect families and clinicians. Communication and Resolution Programs (CRPs) are designed to reduce these negative impacts, rebuild trust, and improve patient safety, but are not consistently implemented. To inform implementation efforts, enable accountability, and promote innovation, it is critical to develop standardized performance measures assessing CRPs’ structure, process, and outcomes. To advance CRP measurement, an interdisciplinary workgroup from the Pathway to Accountability, Compassion, and Transparency (PACT) Leadership and Innovation Network—a group of leading healthcare organizations with CRPs—explores meaningful approaches to measurement and proposes a set of next steps. Interested parties in CRP measurement prioritize developing person-centered outcome and experience measures; assessing equity; addressing clinician and organization concerns about how CRP measurement may affect reputational and legal risk; reducing the burden of measurement; and improving mechanisms for sharing data across organizations to promote transparency, accountability, and broader patient safety improvements. Recommended next steps include: build a national coalition of interested parties to guide the work; overcome barriers to measurement and improve feasibility, especially through the engagement of patient safety and risk management software vendors; explore measure development processes that focus on patient, family, and clinician-centered outcome and experience measures; define nationally recognized standardized CRP measures; consider the role for regulatory and financial incentives to promote their use; and facilitate data sharing and comparative analysis. Ongoing engagement and strategy will be essential to move CRP measurement forward.

## Introduction

Patient harm from healthcare is prevalent and serious (1–3), including: physical injuries or disability; negative impacts on mental health; socio-behavioral consequences such as negative effects on trust, the likelihood of returning for care, or one's ability to work; financial consequences, including the costs of additional healthcare, or loss of income; and in the worst cases, the death of the patient. Such impacts can last for years and these serious safety events often affect families and clinicians (4–7). Leaders and organizations committed to improving patient safety emphasize the need to prevent harm and when harm occurs, improve the response (2, 3). Some healthcare organizations have implemented programs to optimize their responses, commonly referred to as “communication and resolution programs” (CRPs) (although that terminology may evolve, replacing “resolution” with “reconciliation”, acknowledging that harmed people may never experience resolution for their experiences). Such programs have been deemed the “modern ethical paradigm” for responding after harm (8) and are recommended by experts (2, 9). Evidence suggests they may improve a variety of outcomes, including: reducing the emotional impact on patients, regaining their trust, and increasing their willingness to return to or recommend the clinician(s)/organization (10–12); clinicians’ ability to learn and adapt after their patients experience harm (13, 14); organizations’ culture of safety (15), medicolegal experiences and costs (16–21); and the safety of future patients by reducing the risk of event recurrence (22).

Despite the potential of CRPs, evidence suggests most organizations have not implemented them, and those that have may not be applying them consistently (23). While numerous challenges need to be overcome to realize the vision of every organization having a highly-reliable CRP (24), one critical step is to develop and deploy standardized performance measures of CRPs. By assessing CRPs’ structure, process, and outcomes, performance measures can inform organizations’ implementation, internal accountability, continuous learning, and innovation (23, 24). Standardizing measures would facilitate comparative analyses and benchmarking across healthcare organizations, helping identify best practices and allowing for accountability via payors, accrediting and regulatory bodies. In this way, standardized measures are an important part of working towards broader CRP implementation and utilization.

## CRP measurement efforts to-date

The importance of CRP measures has prompted work by researchers (25), a for-profit company promoting CRPs (26), at least one state-based CRP alliance (27), and other non-profit organizations with interests in CRPs and patient safety (23, 24). For example, from 2018 to 2020, sponsored by donations from families whose loved ones died from medical errors, teams at the University of Washington's Collaborative for Accountability for Improvement (CAI) and Ariadne Labs (AL) led a project with national experts to create a comprehensive set of CRP measures. These metrics were tested in several health systems before being shared with the first cohort of the Pathway to Accountability, Compassion, and Transparency (PACT) (28) Collaborative (a group of healthcare organizations implementing CRPs via a learning collaborative model supported by the Institute for Healthcare Improvement, CAI, and AL).

While there are some organizations with CRPs that use some measures, many organizations with CRPs do not routinely measure them, and we are not aware of any organizations with comprehensive CRP measurement systems. There are numerous aspects and dimensions of CRPs that could be measured (examples shown in Table 1). The ambition to comprehensively measure CRPs has been stymied by the challenges of collecting and analyzing data. Those organizations that have succeeded in sustaining some CRP measurement cite a number of benefits, including data shaping CRP processes and implementation; improved dialogue about patient safety and quality improvement; increased engagement of clinicians through constructive conversations about harm events historically concealed by a culture of silence and shame; and enhanced internal accountability for CRPs and safety.

**Table 1:** Aspects, dimensions, and examples of CRP measurement.

Aspects	Example dimensions	Example measures (outcome measures bolded)
Harm events	Volume/severity	# & severity of harm events, # to which the CRP is applied (aka “CRP events”)
	Provenance	How CRP events came to the organization's attention
	Type	Categories of events (e.g., diagnostic delays, medication errors, etc.)
	Locations/services	Places where harm events happened, the involved services
	Equity	Stratifying event-related measures by race, ethnicity, language, etc.
Communication with patients/families	Process-adherence	% patients/families getting initial communication, post-event review communication, and insurer referral when serious harm was preventable
	Timeliness	Time between event reports and initial communications with patients/families; time between completing event reviews and communicating the findings
	Effectiveness & Pt/Fam-centeredness	**Pt/fam assessments of respect, transparency, honesty, compassion, opportunities to ask questions, etc.**
	Equity	Stratifying communication-related measures by race, ethnicity, language, etc.
Support of patients/families	Timeliness	Time between event report, assessments of support needs, offers of support
	Types & Magnitude	Nature & scope of support offered to pt/fam (e.g., counseling, logistics, etc.), % with legal representation or other 3rd party support
	Effectiveness	**Pt/fam assessments of how well their support needs were met**
	Equity	Stratifying support-related measures by race, ethnicity, language, etc.
Support of clinicians	Timeliness	Time between event report, assessments of support needs & offers of support
	Types & magnitude	Nature & scope of support offered to clinicians (e.g., counseling, etc.)
	Effectiveness	**Clinicians’ assessments of how well their support needs were met**
Event review	Pt/fam engagement	% of CRP events that included the pt/fam narrative
	Timeliness	Time between event report and completion of event review
	Contributing factors	Aggregated learnings about the causes of events
	Dissemination	% of findings shared with clinicians and beyond (e.g., via a PSO)
	Outcomes	% CRP events deemed preventable, non-preventable
Patient safety improvements	Timeliness	Time between event report & complete implementation of corrective actions
	Effectiveness	**Corrective action strength, unmitigated contributing factors, # of events**
CRP outcomes	Types	% only involving communication (no litigation or compensation), % involving litigation, % involving a proactive offer of compensation, etc.
	Timeliness	Time between event report & completion of CRP process
	Malpractice experience	# of claims/suits, legal costs, indemnity payments (incl. proportion going to pt/fam who experienced preventable harm), # of NPDB reports
	Effectiveness	**Pt/fam & clinician assessments of the process, degree to which it met their needs, non-physical outcomes (emotional, psychological, financial, and socio-behavioral including trust, healthcare engagement, employment, etc.)**
	Opportunities for improvement	Qualitative information from pt/fam and clinicians
	Equity	Stratifying outcomes by race, ethnicity, language, etc.
CRP resourcing	Team & workload	CRP team roles, credentials, FTE, events per FTE, etc.
	Costs	CRP team FTE, training, supporting systems (e.g., vended software), waived bills, pt/fam & clinician support expenditures, external reviews, legal representation, indemnity payments, etc.

%, percentage; #, number; Pt/fam, patient/family; NPDB, National Practitioner Data Bank. This table represents a sampling of measures noted during the PACT Network Measurement Workgroup's conversations and it is intended to provide context for readers unfamiliar with CRPs. These example measures here have not necessarily been endorsed by PACT.

To advance CRP measurement, the PACT Leadership and Innovation Network—a group of leading healthcare organizations with CRPs, hereafter referred to as the PACT Network—created a Measurement Workgroup in 2023 to explore this issue. The Workgroup identified a set of guiding principles, articulated a strategy for CRP measure development accounting for some of the barriers that hindered prior attempts, proposed an initial set of CRP measures, and developed measurement-related resources that were recently shared with PACT participants for testing. This manuscript has been authored by an interdisciplinary subset of the Workgroup to share what has been learned and a vision for developing CRP measures.

## Interested parties’ perspectives on measurement

Discussions with interested parties, surveys of PACT participants, and recent studies reveal a variety of perspectives about priorities, challenges, and strategies.

Patients and families familiar with the issue of harm from healthcare have been calling for CRP measures that focus on the patient/family experience (29). Evidence suggests CRPs may not always meet patient/family needs (30, 31), so beyond process measures, patient/family-centered outcome and experience measures are crucial to assess whether their needs are being met and to identify aspects of CRPs that may require further development. Some patients—for instance those of lower socio-economic status—are at risk of especially poor experiences and outcomes after healthcare-associated harm (10, 11, 32), making it critical to measure equity. Patient/family advocates also highlight the pitfalls when healthcare organizations are only internally accountable for utilization of a CRP (33). Insufficient access to CRP tools and resources coupled with the lack of a requirement to have CRPs has allowed risk- and legal-oriented process for responding after harm to predominate, thereby adding to the suffering of harmed patients and their families. Public reporting of CRP measures would align with the core values of CRPs—transparency and accountability—and is favored by those who are skeptical about the effectiveness of accrediting and regulatory agencies.

Clinicians involved in harm events want patients and families to receive the care, communication, and support they need, and are concerned about their own experiences. A wide variety of serious consequences can affect clinicians, including emotional and psychological stress, negative effects on their reputation, negative effects on their licensure or credentialing, and litigation (7), all regardless of fault. The CRP approach to responding after harm is designed to mitigate these risks, promote patient safety, and create opportunities for clinicians to communicate and potentially reconcile with harmed patients and their families, which is forbidden in the traditional “deny and defend” approach to harm events. Despite this, many clinicians remain concerned about how CRPs could affect their liability, malpractice premiums, and National Practitioner Data Bank (NPDB) reporting (8), making them interested in measures assessing CRPs’ effects on these outcomes.

Patient safety, patient relations, and risk management professionals are perhaps closer to CRP measurement than other interested parties: they are responsible for identifying “CRP events”—those for which the CRP process should be initiated—and for ensuring each aspect of the CRP process is conducted appropriately, effectively, equitably, and in a person-centered and timely manner. CRP structural and process measures directly assess these professionals’ work, so it is important to understand this group's interests and concerns. These professionals—along with claims, insurance, and legal professionals—likely share the same interests in CRP measures as the clinicians and healthcare organizations they represent. Measurement feasibility is especially important for this group as they manage numerous safety events in complex, high-stakes environments. Unfortunately, when implementing CRPs, many of these professionals discover existing systems for tracking and managing harm events are not optimized for supporting or measuring CRPs, leading most organizations that track CRP measures to do so in spreadsheets adjacent to patient safety and risk management software. Lastly, because there is significant heterogeneity amongst organizations, their contexts, and how they operationalize CRPs, professionals may perceive overly specific CRP measures as misguided.

Healthcare organizations and their senior leaders tend to share the perspectives of their clinicians and have particular interest in their organizations’ reputations and the accrediting, regulatory, and financial consequences of CRPs. Even when organizations want to improve their responses after harm, they may oppose CRP measure requirements because they anticipate challenges resourcing CRP measurement. Some organizations also fear that quantifying the number of harm events suffered by their patients may injure their reputation and may make those data a target of plaintiff attorneys or records requests (for public institutions), thereby increasing legal activity and costs. Even in organizations that believe the public is aware of the prevalence of harm in healthcare and feel less vulnerable to data discovery, risk and legal professionals may cite limited financial resources and increasingly expensive malpractice trends as they counsel organizational resistance to sharing data without protections.

## Next steps

We believe there is an urgency to develop measures that support broader, more consistent implementation of CRPs. While growing the evidence and further optimizing CRPs is important, enough is known to build an initial standardized CRP measure set that can be deployed and refined over time. We propose several strategic steps, summarized in Table 2 and described in detail below.

**Table 2:** Strategy for developing standardized CRP measures.

Strategy	Details
Build a coalition of interested parties	Patient/family advocates; clinicians; patient safety, patient relations, risk management, claims, insurance, and legal professionals; healthcare organization leaders; CRP experts; organizations that have been collecting CRP measures; healthcare measurement experts; PSOs; healthcare accreditation organizations; public and private payers; patient safety and risk management software vendors; health information technology regulatory entities; data collection and management entities; other professional associations, societies, state-level groups (such as the Betsy Lehman Center in Massachusetts), legislators, patient safety advocacy groups; national quality measurement-focused organizations.
Address the barriers to measurement	Reduce the burden of CRP measurement; co-design with patient safety and risk management software vendors; consider data entry & management, data analysis, and the visual display of results; flexibility across care settings; the role for artificial intelligence, large language models, and requirements/regulations to incentivize vendors to better support CRP measurement.
Consider measure development processes	Develop guiding principles, a holistic and aspirational vision of CRP measurement that attends to a variety of measure types—emphasizing outcome and experience measures focused on patient safety and the people affected by events, and using process measures to “diagnose” sub-optimal outcomes and inform CRP implementation—and formal measure development processes.
Define a nationally recognized standardized CRP measure set	A nationally recognized standardized CRP measure set, including a standard definition for “CRP events,” would facilitate integration into patient safety and risk management software, help organizations attest to the Centers for Medicare & Medicaid Services (CMS) Patient Safety Structural Measure (PSSM), inform future patient safety process or outcome measures, and form the foundation of a CRP accreditation program and the basis for financial incentives for organizations.
Develop methods of sharing data amongst organizations	Consider the role for Patient Safety Organizations (PSOs), including the potential benefits, drawbacks, and pitfalls. Once sharing mechanisms are established, conduct comparative analyses and benchmarking, and celebrate CRP measurement successes.

First, build a coalition of interested parties to guide the priorities for measuring CRP programs. While the PACT Network Measurement Workgroup is an example of a coalition that includes all of the aforementioned interested parties, a broader coalition might be more effective by including the perspectives of additional CRP program experts, organizations that have been collecting CRP measures, healthcare measurement experts, PSOs, healthcare accreditation organizations (e.g., The Joint Commission, Det Norske Veritas, Leapfrog), public and private payers [including the Centers for Medicare and Medicaid Services (CMS)], patient safety and risk management software vendors, health information technology entities (e.g., the Office of the National Coordinator), and the Centers for Disease Control (whose National Healthcare Safety Network system will be used to collect data for CMS's Patient Safety Structural Measure). Other professional associations, societies, state-level groups (such as the Betsy Lehman Center in Massachusetts), legislators, and patient safety advocacy groups also play important roles. An existing national quality measurement-focused organization like the Agency for Healthcare Research and Quality (AHRQ) or the National Quality Forum (NQF) could convene such a coalition and guide its work.

Second, the coalition must address the barriers to measurement. Prior efforts have produced lists of CRP measures but have not resulted in widespread, sustained, comprehensive measurement. We believe the primary barrier has been the burden of operationalizing CRP measures. Beyond CRPs, the issue of healthcare quality and safety measurement burden is significant (34), and experts have recommended reducing the number of measures, facilitating collection and analysis, leveraging digital technologies, and including cost-effectiveness as key considerations in measurement development and selection processes (35). These same principles should be applied to CRP measurement. Without solving the feasibility issue, we can expect the status quo of minimal CRP measurement to persist. Engaging patient safety and risk management software vendors early in the measure development process to facilitate testing and optimize product design will be critical. Professionals’ CRP-related workflows must be intuitive and efficient to alleviate the burden of collecting and analyzing data. It will be important to consider how these workflows and CRP measures may vary across healthcare settings. This will require attention to data entry and management processes, data analysis, and visual display of the results. Artificial intelligence and large language models could help identify CRP events, reduce the burden of documentation and data entry, or otherwise facilitate CRP measurement. The coalition should carefully consider the role of requirements or regulations to incentivize such companies to incorporate CRP data elements and measures into their products, as has been done for electronic health records (36).

Third, the coalition should consider the processes it will use to develop and prioritize measures. Developing and adopting a set of guiding principles—for example the Global Principles for Measuring Patient Safety (35)—could facilitate decisions when interested parties’ perspectives conflict. Developing consensus around a comprehensive system of CRP measurement—including structural, process, outcome, balancing, and qualitative measures that attend to all the aspects and dimensions of CRPs (Table 1), and promoting improvement and accountability—could help align interested parties. Such an aspirational vision would need to be balanced by a pragmatic, phased approach. The PACT Network Measurement Workgroup found that a small number of measures focused on the context of organizations’ CRPs, the composition of their CRP teams, and the initial stage of the CRP process, resonated well with a broad variety of healthcare organizations. Reliably initiating the CRP process is critical to the effectiveness of CRPs, and organizations newly implementing a CRP begin with the initial phase, so this strategy maximizes overlap amongst organizations. These measures are now being tested and a guide with data element and measure definitions for the initial stage of the CRP process is publicly available (37).

Alternatively, a strategy prioritizing outcome and experience measures—selectively using process measures to “diagnose” sub-optimal outcomes—may ultimately better drive improvement and would align with contemporary measurement strategies (38). Outcome measures should include assessments of patient safety since preventing harm is always preferrable to having to respond after it has occurred. Experience measures should avoid the construct of satisfaction—which is misaligned with the deeply dissatisfying experience of a recent harm event—instead focusing on constructs such as respect, honesty, quality of communication, compassion, fairness, trust, and well-being (29). These could be assessed by combining standardized surveys that produce quantitative scores with comments or other qualitative data, all collected using digital technology. Measure developers would need to carefully consider the timing, provenance, and coordination of post-harm event surveys as relates to CRP processes (39). It is important that patient safety professionals and healthcare organization leaders recognize the inherent limitations of standardized measures when it comes to understanding peoples’ complex experiences after harm. To mitigate these limitations, digitally collected CRP measures should regularly be supplemented with direct patient, family, and clinician engagement.

In addition to the measures developed by the PACT Network Measurement Workgroup, we have also encouraged interested and engaged PACT organizations to share any additional measures they are using so that other organizations can learn from them. Despite this encouragement, we have observed little innovation. As a result, we suspect more formal processes will be needed to move the field forward: securing funding for the work, engaging measure developers, building consensus about a vision for CRP measurement, proactively attending to barriers, and selecting healthcare organizations to test measures.

Fourth, the coalition should work towards measure standardization, beginning with the definition of what constitutes a “CRP event.” Published reports assessing CRPs’ effectiveness have used a range of definitions, from simple [e.g., “unexpected adverse outcomes” (18)] to more complex, including events: exceeding a pre-specified level of harm (e.g., moderate, temporary or worse); requiring additional unplanned care (e.g., transfer to the ICU, prolonged length of stay, an unplanned invasive procedure or several ambulatory visits); meeting external reporting criteria; for which the patient/family report serious harm (regardless of the organization's severity score); for which the patient, family, or a clinician requests the CRP; or involving a claim or litigation (20, 37). A standardized CRP event definition would ensure a consistent denominator for comparative analysis amongst organizations. Consensus building processes to define CRP events are likely most feasible in the near-term, but future research could provide formative insights through more rigorous evaluation of the characteristics and performance of various CRP event definitions.

After sufficient measure testing, the coalition could identify a nationally recognized standardized CRP measure set. This would facilitate the process of incorporating measures into patient safety and risk management software. Such a measure set could help organizations attest to CMS's recently developed Patient Safety Structural Measure: “*Our hospital uses standard measures to track the performance of our communication and resolution program, and reports these measures to the governing board at least quarterly.”* (40) In the future, CMS or other entities could incorporate standardized CRP measures into required patient safety measure sets. Such measures could also form the foundation of a CRP accreditation program. Public reporting of standardized CRP measures could help patients/families make informed choices about their care, and it might also help healthcare organizations rebuild trust with their communities. Publicly reporting process measures might be more acceptable to healthcare organizations than outcome measures, which may be more easily shared through patient safety organizations (PSOs).

Lastly, standardized measure sets, such as those produced by the Core Quality Measures Collaborative convened by NQF, have been used in value-based purchasing and Alternate Payment Models (41). The use of unvalidated or inaccurate quality measures to reduce reimbursement for poor performance is problematic, so before considering CRP measures for such measure sets, they would need to be rigorously evaluated. If validated as accurate measures of quality, then in close collaboration with quality measurement organizations, payors, lawmakers and policymakers, perhaps organizations that demonstrated highly reliable CRPs could be awarded financial benefits or spared penalties. As valuable as standardized measures will be, they should evolve, so ongoing processes for learning, innovation, and updates will be important.

Fifth, a widely used nationally recognized standardized CRP measure set would unlock the potential for comparative analysis or benchmarking, but that would require systems for sharing and comparing data amongst organizations. PSOs could be a way of doing so but have numerous challenges including limited participation (42). To be consistent with the ethical principles of CRPs, the use of PSOs would need to promote transparency, accountability, patient safety improvements, and optimal responses to patients and families after harm. Once sharing mechanisms are established, methods of comparative analysis could be developed and refined, ultimately using them to identify top performers, drive innovation, and promote uptake of best practices. Methods for celebrating measurement successes, whether early adopters of CRP measures, innovators, or top performers, may help demonstrate the value of CRP measures to interested parties.

## Conclusion

While the development and deployment of CRP measures will take an interdisciplinary effort, doing so is a critical part of improving patient safety, the quality of care, and the experience of everyone affected when patients are harmed by healthcare. The outcomes of patients, families, and clinicians should be the focus of CRP measurement, but additional aspects and dimensions of CRPs will require assessment. Despite the clear rationale and numerous benefits of CRP measures, healthcare organizations may not readily collect, analyze, or share their data. A strategic approach to developing CRP measures is essential to move beyond the status quo of minimal CRP measurement. Ongoing engagement of organizational leaders to demonstrate how CRPs are essential components of their clinical mission and the work environment for their clinicians, along with regulatory, accrediting, and/or financial incentives, will likely be necessary.

## Data Availability

The original contributions presented in the study are included in the article/Supplementary Material, further inquiries can be directed to the corresponding author.

## References

[1] Bates DW Levine DM Salmasian H Syrowatka A Shahian DM Lipsitz S The safety of inpatient health care. N Engl J Med. (2023) 388(2):142–53. 10.1056/NEJMsa2206117 36630622

[2] National Action Plan to Advance Patient Safety. IHI—Institute for Healthcare Improvement. Boston, MA: Institute for Healthcare Improvement (2020). Available online at: https://www.ihi.org:443/Engage/Initiatives/National-Steering-Committee-Patient-Safety/Pages/National-Action-Plan-to-Advance-Patient-Safety.aspx (cited September 29, 2023)

[3] World Health Organization. Global Patient Safety Report 2024. Geneva: World Health Organization (2024). Available online at: https://www.who.int/publications/i/item/9789240095458

[4] Ottosen MJ Sedlock EW Aigbe AO Bell SK Gallagher TH Thomas EJ. Long-Term impacts faced by patients and families after harmful healthcare events. J Patient Saf. (2018) 17(8):e1145–51. 10.1097/PTS.0000000000000451 PMC6050155 29346175

[5] Betsy Lehman Center. The Financial and Human Cost of Medical Error.. and how Massachusetts can Lead the way on Patient Safety. Boston, MA: Betsy Lehman Center for Patient Safety (2019). Available online at: https://www.betsylehmancenterma.gov/assets/uploads/Cost-of-Medical-Error-Report-2019.pdf

[6] NORC at the University of Chicago and IHI/NPSF Lucian Leape Institute. Americans’ Experiences with Medical Errors and Views on Patient Safety. Chicago, IL: Institute for Healthcare Improvement (2017). Available online at: http://www.ihi.org/about/news/Documents/IHI_NPSF_NORC_Patient_Safety_Survey_2017_Final_Report.pdf

[7] Wu AW Shapiro J Harrison R Scott SD Connors C Kenney L The impact of adverse events on clinicians: what’s in a name? J Patient Saf. (2020) 16(1):65–72. 10.1097/PTS.0000000000000256 29112025

[8] Gallagher TH Kachalia A. Responding to medical errors—implementing the modern ethical paradigm. N Engl J Med. (2024) 390(3):193–7. 10.1056/NEJMp2309554 38226840

[9] President’s Council of Advisors on, Science and Technology. A Transformational Effort on Patient Safety. Washington, DC: Office of Science and Technology Policy, Executive Office of the President of the United States (2023). Available online at: https://www.whitehouse.gov/wp-content/uploads/2023/09/PCAST_Patient-Safety-Report_Sept2023.pdf

[10] Prentice JC Bell SK Thomas EJ Schneider EC Weingart SN Weissman JS Association of open communication and the emotional and behavioural impact of medical error on patients and families: state-wide cross-sectional survey. BMJ Qual Saf. (2020) 29(11):883–94. 10.1136/bmjqs-2019-010367 31959717

[11] Sokol-Hessner L Dechen T Folcarelli P McGaffigan P Stevens JP Thomas EJ Associations between organizational communication and Patients’ experience of prolonged emotional impact following medical errors. Jt Comm J Qual Patient Saf. (2024) 50(9):620–9. 10.1016/j.jcjq.2024.03.002 38565471

[12] Fisher KA Gallagher TH Smith KM Zhou Y Crawford S Amroze A Communicating with patients about breakdowns in care: a national randomised vignette-based survey. BMJ Qual Saf. (2020) 29(4):313–9. 10.1136/bmjqs-2019-009712 31723017 PMC7170008

[13] Plews-Ogan M May N Owens J Ardelt M Shapiro J Bell SK. Wisdom in Medicine Acad Med J Assoc Am Med Coll. (2016) 91(2):233–41. 10.1097/ACM.0000000000000886 26352764

[14] Etchegaray JM Gallagher TH Bell SK Dunlap B Thomas EJ. Error disclosure: a new domain for safety culture assessment. BMJ Qual Saf. (2012) 21(7):594–9. 10.1136/bmjqs-2011-000530 22562878

[15] de Carvalho REFL Bates DW Syrowatka A Almeida I Sousa L Goncalves J Factors determining safety culture in hospitals: a scoping review. BMJ Open Qual. (2023) 12(4):e002310. 10.1136/bmjoq-2023-002310 37816540 PMC10565149

[16] Kachalia A Kaufman SR Boothman R Anderson S Welch K Saint S Liability claims and costs before and after implementation of a medical error disclosure program. Ann Intern Med. (2010) 153(4):213–21. 10.7326/0003-4819-153-4-201008170-00002 20713789

[17] Adams MA Elmunzer BJ Scheiman JM. Effect of a health system’s medical error disclosure program on gastroenterology-related claims rates and costs. Am J Gastroenterol. (2014) 109(4):460–4. 10.1038/ajg.2013.375 24698856

[18] Lambert BL Centomani NM Smith KM Helmchen LA Bhaumik DK Jalundhwala YJ The “seven pillars” response to patient safety incidents: effects on medical liability processes and outcomes. Health Serv Res. (2016) 51(Suppl 3):2491–515. 10.1111/1475-6773.12548 27558861 PMC5134341

[19] Mello MM Greenberg Y Senecal SK Cohn JS. Case outcomes in a communication-and-resolution program in New York hospitals. Health Serv Res. (2016) 51(Suppl 3):2583–99. 10.1111/1475-6773.12594 27781266 PMC5134351

[20] Mello MM Kachalia A Roche S Niel MV Buchsbaum L Dodson S Outcomes in two Massachusetts hospital systems give reason for optimism about communication-and-resolution programs. Health Aff. (2017) 36(10):1795–803. 10.1377/hlthaff.2017.0320 28971925

[21] Kachalia A Sands K Niel MV Dodson S Roche S Novack V Effects of a communication-and-resolution program on hospitals’ malpractice claims and costs. Health Aff. (2018) 37(11):1836–44. 10.1377/hlthaff.2018.0720 30395501

[22] Brenner MJ Hickson GB Rushton CH Prince MEP Bradford CR Boothman RC. Honesty and transparency, indispensable to the clinical mission—part II. Otolaryngol Clin North Am. (2022) 55(1):63–82. 10.1016/j.otc.2021.07.018 34823721

[23] Gallagher TH Mello MM Sage WM Bell SK McDonald TB Thomas EJ. Can communication-and-resolution programs achieve their potential? Five key questions. Health Affairs. (2018) 37(11):1845–52. 10.1377/hlthaff.2018.0727 30395493

[24] Gallagher TH Boothman RC Schweitzer L Benjamin EM. Making communication and resolution programmes mission critical in healthcare organisations. BMJ Qual Saf. (2020) 29(11):875–8. 10.1136/bmjqs-2020-010855 32371456

[25] Schulz-Moore JS Bismark M Jenkinson C Mello MM. Assessing Patients’ experiences with medical injury reconciliation processes: item generation for a novel survey questionnaire. Jt Comm J Qual Patient Saf. (2021) 47(6):376–84. 10.1016/j.jcjq.2021.03.004 33836941

[26] BETA Healthcare Group and HQI. BETA HEART® for HQI Members—BETA HEART® Guideline. Alamo, CA: BETA Healthcare Group and HQI (2021). Available online at: https://hqinstitute.org/wp-content/uploads/sites/5/2022/08/hqi_cares_-_beta_heart_guideline_071521_jhc_edits.pdf (cited July 18, 2024)

[27] Betsy Lehman Center for Patient Safety. Metrics Guidance—Communication, Apology and Resolution (CARe). Boston, MA: Betsy Lehman Center for Patient Safety (2023). Available online at: https://betsylehmancenterma.gov/assets/uploads/CARe_MetricsGuidance.pdf (cited July 16, 2024)

[28] PACT: Pathway to Accountability, Compassion, and Transparency. Boston, MA: Ariadne Labs (2024). Available online at: https://www.ariadnelabs.org/pact/ (cited July 17, 2024)

[29] Kachalia A Hemmelgarn C Gallagher TH. Communication after medical error: the need to measure the patient experience. Jt Comm J Qual Patient Saf. (2024) S1553-7250(24):00202–2. 10.1016/j.jcjq.2024.06.006 39013757

[30] Moore J Bismark M Mello MM. Patients’ experiences with communication-and-resolution programs after medical injury. JAMA Intern Med. (2017) 177(11):1595–603. 10.1001/jamainternmed.2017.4002 29052704 PMC5710270

[31] Moore J Mello MM. What patients expect and deserve—reply. JAMA Intern Med. (2018) 178(4):578–9. 10.1001/jamainternmed.2018.0135 29610888

[32] Olazo K Gallagher TH Sarkar U. Experiences and perceptions of healthcare stakeholders in disclosing errors and adverse events to historically marginalized patients. J Patient Saf. (2023) 19(8):547–52. 10.1097/PTS.0000000000001173 37921753

[33] Handley GM. Disclosure programmes in the US—an inadequate response to medical error. BMJ. (2024) 385:q1318. 10.1136/bmj.q1318 38876491

[34] Saraswathula A Merck SJ Bai G Weston CM Skinner EA Taylor A The volume and cost of quality metric reporting. JAMA. (2023) 329(21):1840–7. 10.1001/jama.2023.7271 37278813 PMC10245189

[35] IHI Lucian Leape Institute. The Salzburg Statement on Moving Measurement into Action: Global Principles for Measuring Patient Safety. Boston, MA: Salzburg Global Seminar & Institute for Healthcare Improvement (2019). Available online at: https://www.salzburgglobal.org/fileadmin/user_upload/Documents/2010-2019/2019/Session_622/SalzburgGlobal_Statement_622_Patient_Safety_01.pdf (cited April 6, 2024)

[36] The Office of the National Coordinator for Health Information Technology. Strategy on Reducing Burden Relating to the Use of Health IT and EHRs. Washington, DC: Assistant Secretary for Technology Policy, U.S. Department of Health and Human Services (2020). Available online at: https://www.healthit.gov/topic/usability-and-provider-burden/strategy-reducing-burden-relating-use-health-it-and-ehrs (cited July 23, 2024)

[37] Pathway to Accountability, Compassion, and Transparency (PACT). PACT CRP Measurement Guide. Boston, MA: Pathway to Accountability, Compassion, and Transparency (2024). Available online at: https://www.ariadnelabs.org/wp-content/uploads/2024/08/PACT-Measurement-Guide-Public-2024-08-17.pdf (cited August 20, 2024)

[38] Jacobs DB Schreiber M Seshamani M Tsai D Fowler E Fleisher LA. Aligning quality measures across CMS — the universal foundation. N Engl J Med. (2023) 388(9):776–9. 10.1056/NEJMp2215539 36724323

[39] Hudon C Fortin M Haggerty JL Lambert M Poitras ME. Measuring Patients’ perceptions of patient-centered care: a systematic review of tools for family medicine. Ann Fam Med. (2011) 9(2):155–64. 10.1370/afm.1226 21403143 PMC3056864

[40] Centers for Medicare & Medicaid Services. Patient Safety Structural Measure (PSSM). Baltimore, MD: Centers for Medicare & Medicaid Services (2024). p. 1255–345. Available online at: https://www.federalregister.gov/public-inspection/2024-17021/medicare-medicaid-and-childrens-health-insurance-programs-hospital-inpatient-prospective-payment (cited August 5, 2024)

[41] NQF: CQMC Core Sets. Available online at: https://www.qualityforum.org/CQMC_Core_Sets.aspx (cited July 17, 2024)

[42] U.S. Department of Health and Human Services Office of Inspector General. Patient Safety Organizations: Hospital Participation, Value, and Challenges. Washington, DC: U.S. Department of Health and Human Services (2019). Available online at: https://oig.hhs.gov/oei/reports/oei-01-17-00420.pdf (cited August 7, 2024)

